# Development & validation of the BSI-9: A brief screening tool for the SAD Triad

**DOI:** 10.1192/j.eurpsy.2021.1077

**Published:** 2021-08-13

**Authors:** C. Macdonald, K. Brophy, A. Coroiu, E. Braehler, A. Korner

**Affiliations:** 1 Educational And Counselling Psychology, McGill University, Montreal, Canada; 2 Social And Behavioural Sciences, Harvard T.H. Chan School of Public Health, Boston, United States of America; 3 Psychology, Medical Center of the University of Leipzig, Leipzig, Germany

**Keywords:** Scale development, Brief Symptom Inventory, Factor structure, Psychological Distress

## Abstract

**Introduction:**

The Brief Symptom Inventory (BSI-53) was originally developed as a shorter alternative to the Symptom Checklist-90R, which captures a breath of psychopathology. Subsequently, the BSI-53 was further streamlined to an 18-item scale assessing psychological distress in terms of somatization (S), anxiety (A), and depression (D) – also known as the “SAD Triad”. The BSI-18 has been shown to have good validity in the German general population.

**Objectives:**

The objective of the present study was to further improve the ease of use of the BSI as a clinical screening tool by developing a reliable and valid 9-item version of the BSI-18.

**Methods:**

A representative sample of the German general population (N=2,516) was surveyed for demographic information and completed a variety of questionnaires, including the BSI-18. Confirmatory factor analyses, item-level statistics, and correlations were used to select three rather heterogeneous items for each subscale and confirm the model fit.

**Results:**

The proposed 3-factor model of the BSI-9, corresponding to the SAD triad, demonstrated a good model fit. The internal consistency (Cronbach’s alpha) was .87 for the total scale, .72 for the somatisation scale, .79 for the depression scale, and .68 for the anxiety scale. Each of the subscales were significantly related to the Patient Health Questionnaire-4 and Hopkins Symptoms Checklist-25 in the hypothesized direction.

**Conclusions:**

The BSI-9 provides researchers and clinicians with a brief, effective, and valid tool to screen for anxiety, depression, and somatization, thus preventing potential overload for research participants and flagging patients who might need further clinical assessment.

